# The Effects of Online Health Information–Seeking Behavior on Sexually Transmitted Disease in China: Infodemiology Study of the Internet Search Queries

**DOI:** 10.2196/43046

**Published:** 2023-05-12

**Authors:** Xuan Li, Kun Tang

**Affiliations:** 1 Vanke School of Public Health Tsinghua University Beijing China

**Keywords:** sexually transmitted infections, Baidu search index, Baidu search rate, online health information-seeking behavior, long-term effect, effect, disease, internet, prevention, data, treatment, surveillance

## Abstract

**Background:**

Sexually transmitted diseases (STDs) are a serious issue worldwide. With the popularity of the internet, online health information-seeking behavior (OHISB) has been widely adopted to improve health and prevent disease.

**Objective:**

This study aimed to investigate the short-term and long-term effects of different types of OHISBs on STDs, including syphilis, gonorrhea, and AIDS due to HIV, based on the Baidu index.

**Methods:**

Multisource big data were collected, including case numbers of STDs, search queries based on the Baidu index, provincial total population, male-female ratio, the proportion of the population older than 65 years, gross regional domestic product (GRDP), and health institution number data in 2011-2018 in mainland China. We categorized OHISBs into 4 types: concept, symptoms, treatment, and prevention. Before and after controlling for socioeconomic and medical conditions, we applied multiple linear regression to analyze associations between the Baidu search index (BSI) and Baidu search rate (BSR) and STD case numbers. In addition, we compared the effects of 4 types of OHISBs and performed time lag cross-correlation analyses to investigate the long-term effect of OHISB.

**Results:**

The distributions of both STD case numbers and OHISBs presented variability. For case number, syphilis, and gonorrhea, cases were mainly distributed in southeastern and northwestern areas of China, while HIV/AIDS cases were mostly distributed in southwestern areas. For the search query, the eastern region had the highest BSI and BSR, while the western region had the lowest ones. For 4 types of OHISB for 3 diseases, the BSI was positively related to the case number, while the BSR was significantly negatively related to the case number (*P*<.05). Different categories of OHISB have different effects on STD case numbers. Searches for prevention tended to have a larger impact, while searches for treatment tended to have a smaller impact. Besides, due to the time lag effect, those impacts would increase over time.

**Conclusions:**

Our study validated the significant associations between 4 types of OHISBs and STD case numbers, and the impact of OHISBs on STDs became stronger over time. It may provide insights into how to use internet big data to better achieve disease surveillance and prevention goals.

## Introduction

Sexually transmitted diseases (STDs) have been and are still a serious global health issue. According to the World Health Organization, more than 1 million people acquired an STD every day worldwide in the past few years [1]. In China, syphilis, gonorrhea, and AIDS due to HIV are the 3 major STDs that have been listed as category B notifiable infectious diseases according to the Law of the People’s Republic of China on the Prevention and Control of Infectious Diseases [2]. Although highly emphasized, these STDs are still highly prevalent nationwide. According to the statistics of the China Health and Wellness Commission, there were 480,020 cases of newly diagnosed syphilis, 127,803 cases of newly diagnosed gonorrhea, and 60,154 cases of newly diagnosed HIV/AIDS in 2021. Their geographical distributions show significant regional disparities that require scientific attention to properly allocate relevant health care resources [3,4].

The internet has gradually become a primary source of health information worldwide [5]. Approximately 59% of American adults rely on the internet as their primary health information source [6]. In China, it has been estimated that approximately 70.79% of the population frequently uses the internet to seek health information [7]. Online health information–seeking behavior (OHISB) has been demonstrated to be an effective and popular pathway to facilitate human health [8,9]. OHISB can not only improve one’s health knowledge and decision-making ability regarding medical treatment [9,10], but also facilitate one’s preventative participation, health information sharing, and so on [11,12]. However, most relevant studies have been limited to investigations of the effects of OHISB on cognitive and behavioral health in individuals, and few have associated OHISB with disease outcomes at the regional population level.

There are different types of OHISBs for various contexts, purposes, and populations. Some people tended to seek out illness information, such as cancer and chronic illness information, with respect to coping with a health-related threat or guiding medical decision-making, while some tended to seek out wellness information, such as nutrition and exercise information, with respect to adopting preventive behaviors [13,14]. The general motivations for OHISB mainly included self-care, disease prevention, medication, treatment, and so on; such motivations were significantly different among subgroups, such as males and females [15]. For STDs, it was found that internet users mainly searched for symptoms, treatment, and social and emotional opinions [16]. Through offline surveys, a small but increasing number of studies have focused on the effects of different types of health information–seeking behavior. By dividing internet users into different groups, a cross-sectional study found that those who only searched for wellness information tended to have better health status than those who only searched for illness information [17,18]. Another study also built 3 models based on the role of the internet on women’s health to identify the correlation between OHISB and health [18]. However, few studies have compared the effects of different OHISBs based on internet big data.

Baidu was the predominant search engine, with a priority selection rate of 93.1% among Chinese internet users [19]. Its subproduct, the Baidu index, is a public database based on Baidu that records web-based and mobile phone search queries at national and subnational scales [20]. An increasing number of studies have used the Baidu index to build predictive models for geographical disease surveillance, especially for STDs [21,22]. However, to date, few studies have linked the Baidu index with OHISBs to investigate specific effects on disease outcomes. Since the outbreak of the COVID-19 pandemic, some studies have investigated the time lag effect of the Baidu index on the number of COVID-19 cases [23]. However, for other diseases, such as STDs, little attention has been given to the long-term health effects associated with Baidu index data. In addition, almost all of the related studies used only a single data variable from the Baidu index and did not take socioeconomic or local medical conditions into consideration.

Accordingly, we designed a study with 3 objectives. First, based on Baidu search behavior, we sought to identify the effects of OHISB on the case numbers of STDs, including syphilis, gonorrhea, and HIV/AIDS, at the regional population level in China. Second, by controlling for the effects of local economic and medical conditions, we aimed to compare the effects of different types of OHISBs on disease outcomes. Third, based on yearly time series data sets, we investigated the long-term effect of different OHISBs on STDs.

## Methods

### Real-World Databases

To explore the relationships between search queries and disease incidence, we collected epidemiological data on monthly confirmed cases of incident syphilis, gonorrhea, and HIV/AIDS in all 31 provinces in mainland China from January 2011 to December 2018; these data were obtained from the Chinese Center for Disease Control and Prevention’s nationwide disease reporting system. In addition, to control for the confounding effects of socioeconomic and medical factors, we collected yearly total population, male-female ratio, the proportion of the population older than 65 years, gross regional domestic product (GRDP), and health institution number data from the same regions and time period as above, obtained from the statistical yearbook of the National Bureau of Statistics of China. The GRDP per capita and the number of health institutions per capita were then calculated by dividing these numbers by the total population. According to the official criteria for economic regional division, we also divided mainland China into 4 major economic regions: the eastern, central, western, and northeastern regions. Among them, the eastern region had the highest income level, while the western region had the lowest income level. The list of each region and its provinces or municipalities is shown in Table S1 in Multimedia Appendix 1 [24].

### Search Query Databases

For each keyword, the Baidu search index (BSI) was calculated as the weighted sum of search frequencies based on the search volume of internet users using the Baidu search engine. In addition, we defined the Baidu search rate (BSR) as the ratio of the BSI to the total population to reflect the number of searches per person for a particular disease in the locality. In this study, we collected daily BSI and BSR data for each disease in all 31 provinces in mainland China from January 1, 2011, to December 31, 2018.

According to the purpose and content, based on previous studies and Baidu characteristics, we divided OHISBs into 4 categories: concept, symptoms, treatment, and prevention. Concept-related search terms were mainly name, definition, and abbreviation, which allowed searchers to obtain a more comprehensive understanding of the concept of the disease. Symptom-related search terms were mainly the potential manifestations of the disease. Treatment-related search terms mainly focused on treatment options, the effectiveness of treatment and drugs, and the curability of the disease. Prevention-related search terms are mainly focused on preventive medicine issues, such as the incubation period, route of transmission, and infectivity of the disease. The search strategy is shown in Table S2 in Multimedia Appendix 1.

We investigated more than 1 keyword for each category so that the effects of extreme values and noisy data could be largely reduced through the combination of multiple results. Thus, for each disease, we used principal component analysis to combine the multiple search terms under the same topic.

### Statistical Analyses

Analyses were conducted in R (version 4.0.3; R Core Team and the R Foundation for Statistical Computing). For both real-world case data and search query data, we calculated descriptive statistics, comprising the mean and SD of yearly confirmed cases and the median and IQR of yearly BSI and BSR under each topic among 4 major economic regions. A 1-way ANOVA or Kruskal-Wallis test was applied to examine differences according to normal or abnormal distribution. A 2-tailed *P* value <.05 was considered statistically significant. All descriptive analysis was run by the R package “*tableone.*”

A multiple linear regression model was constructed to explore correlations between search query data and case data. All data were standardized so that the model coefficients were comparable. For each search term category, we examined the association between the number of disease cases and BSI and the number of disease cases and BSR before and after controlling for socioeconomic and medical conditions to analyze the sensitivity and guarantee the stability of the results. In addition, time lag cross-correlation analyses were performed by regression models to investigate the lag effects of the BSI and BSR on disease case numbers. Lag periods ranging from 1 to 7 years were evaluated, with a new data set created for each lag period that included the BSI and BSR values along with the corresponding disease case numbers occurring after the lag period.

### Ethical Considerations

This study used 2 public databases that were open for public health research, and we adhered to local, national, regional, and international laws and regulations regarding the protection of personal information, privacy, and human rights. No data involved any personal information.

## Results

### Distribution of STDs and OHISBs

**Figure 1:**
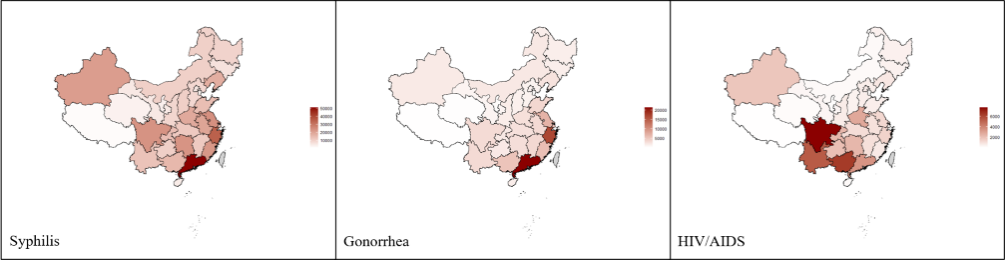
Provincial distributions of average yearly numbers of cases of syphilis, gonorrhea, and HIV/AIDS from 2011 to 2018 in mainland China.

Since 2011, China has had an average of 434,252 new cases of syphilis, 109,027 new cases of gonorrhea, and 46,983 new cases of HIV/AIDS each year. The national trends in yearly numbers of confirmed cases of these 3 diseases are shown in Figure S1 in Multimedia Appendix 1, with increasing trends annually. Figure 1 shows the provincial distributions of average yearly numbers of confirmed cases of these 3 diseases, with variability across the nation. Syphilis and gonorrhea were mainly distributed in the eastern region (a provincial average of 17,999.41 for syphilis and 6425.50 for gonorrhea per year), while HIV/AIDS was mainly distributed in the western region (a provincial average of 2266.05).

OHISBs in Baidu across the nation also showed variability. Table 1 shows the regional yearly BSIs and BSRs for the 4 OHISB categories for syphilis, gonorrhea, and HIV/AIDS, which were standardized through the principal component analysis algorithm. It can be inferred that the BSI and BSR for each disease were significantly different among the 4 economic regions. The provinces in the eastern region had the highest BSI and BSR among the 4 OHISB categories, except for the BSR for gonorrhea treatment. The provinces in the western region had the lowest BSI, and the provinces in the northeastern region had the lowest BSR.

**Table 1:** Regional performance of yearly BSI^a^ and BSR^b^ of 4 categories of syphilis, gonorrhea, and HIV/AIDS at the provincial level from 2011 to 2018.

Disease and category		BSI										BSR							
		Eastern, median (IQR)	Central, median (IQR)		Western, median (IQR)		Northeastern, median (IQR)		*P* value		Eastern, median (IQR)			Central, median (IQR)	Western, median (IQR)		Northeastern, median (IQR)		*P* value
**Syphilis**																			
	Concept	1.04 (–0.47 to 3.04)	–0.05 (–0.94 to 0.90)	–1.21 (–1.96 to –0.34)		–0.22 (–0.75 to 0.27)		<.001		0.53 (–0.72 to 1.69)			–0.66 (–1.34 to –0.16)		–0.37 (–1.12 to 0.27)	0.20 (–0.70 to 0.59)		<.001	
	Symptoms	0.51 (–0.90 to 2.65)	0.18 (–1.26 to 1.08)	–1.26 (–2.02 to –0.15)		0.22 (–1.02 to 0.95)		<.001		–0.01 (–1.19 to 1.76)			–0.91 (–1.53 to –0.15)		–0.53 (–1.31 to 0.82)	0.02 (–1.06 to 1.16)		.002	
	Treatment	0.54 (–0.80 to 1.90)	0.09 (–1.00 to 0.93)	–1.06 (–1.87 to 0.28)		0.77 (–0.42 to 1.26)		<.001		–0.21 (–1.02 to 1.08)			–0.80 (–1.30 to –0.08)		–0.40 (–1.27 to 0.82)	0.33 (–0.49 to 1.24)		.003	
	Prevention	0.66 (–0.48 to 1.74)	0.18 (–0.81 to 0.84)	–0.79 (–1.57 to 0.07)		0.43 (–0.46 to 0.93)		<.001		–0.10 (–0.75 to 0.96)			–0.57 (–1.05 to –0.09)		–0.25 (–0.94 to 0.56)	0.34 (–0.54 to 0.84)		.001	
**Gonorrhea**																			
	Concept	0.88 (–0.38 to 2.44)	–0.07 (–0.62 to 0.74)	–1.25 (–1.96 to –0.39)		–0.27 (–0.52 to 0.49)		<.001		0.50 (–0.64 to 1.56)			–0.58 (–1.11 to –0.17)		–0.37 (–1.01 to 0.22)	0.30 (–0.06 to 0.65)		<.001	
	Symptom	0.65 (–0.03 to 1.88)	0.24 (–0.32 to 0.49)	–0.88 (–1.75 to –0.25)		–0.08 (–0.37 to 0.38)		<.001		0.52 (–0.60 to 1.77)			–0.70 (–0.95 to –0.30)		–0.21 (–0.65 to 0.33)	0.20 (–0.05 to 0.58)		<.001	
	Treatment	1.07 (–0.13 to 2.27)	0.14 (–0.68 to 0.82)	–1.15 (–2.64 to –0.04)		0.59 (0.13 to 1.30)		<.001		–0.05 (–1.06 to 2.25)			–0.91 (–1.31 to –0.43)		–0.19 (–0.97 to 0.73)	0.90 (0.33 to 1.79)		<.001	
	Prevention	0.76 (0.03 to 1.34)	–0.17 (–0.50 to 0.52)	–0.85 (–1.22 to –0.28)		0.07 (–0.32 to 0.56)		<.001		–0.02 (–0.43 to 1.26)			–0.43 (–0.71 to –0.05)		–0.43 (–0.75 to 0.06)	0.27 (–0.23 to 0.59)		<.001	
**HIV/AIDS**																			
	Concept	0.83 (–0.61 to 1.94)	0.21 (–0.83 to 1.15)	–0.83 (–1.70 to 0.24)		–0.33 (–1.08 to –0.05)		<.001		0.09 (–0.96 to 1.47)			–0.68 (–1.35 to –0.08)		–0.01 (–1.01 to 0.65)	–0.08 (–1.10 to 0.24)		.005	
	Symptom	0.16 (–0.70 to 1.92)	–0.09 (–0.98 to 1.07)	–1.02 (–1.58 to 0.03)		–0.67 (–1.23 to 0.32)		<.001		–0.04 (–1.03 to 1.12)			–0.87 (–1.32 to –0.05)		–0.31 (–0.99 to 0.90)	–0.57 (–1.05 to 0.27)		.003	
	Treatment	1.09 (–0.22 to 3.20)	–0.15 (–0.76 to 1.05)	–1.24 (–2.51 to 0.46)		–0.31 (–0.81 to 0.06)		<.001		0.28 (–0.85 to 1.95)			–0.70 (–1.35 to –0.31)		–0.18 (–1.19 to 0.38)	0.10 (–0.80 to 0.75)		<.001	
	Prevention	0.52 (–0.63 to 1.47)	0.10 (–0.68 to 0.81)	–0.71 (–1.37 to 0.09)		–0.11 (–0.81 to 0.26)		<.001		–0.09 (–0.83 to 0.92)			–0.67 (–1.15 to –0.27)		0.06 (–0.79 to 0.62)	0.03 (–0.88 to 0.33)		<.001	

^a^BSI: Baidu search index.

^b^BSR: Baidu search rate.

### Correlation Analyses Between OHISB and STDs

We constructed a multiple linear regression model to explore the correlations between the BSIs and BSRs of the 4 OHISB categories and the real-world numbers of cases for the 3 diseases at the provincial level, the results of which are shown in Table 2. The results of the 3 models showed that the correlation results were stable and similar before and after controlling for socioeconomic and medical conditions. The BSIs for all 4 categories were significantly positively correlated with the number of cases of the 3 diseases, while the BSRs for the 4 categories mostly had significantly negative correlations with the number of disease cases. Thus, the more times a province searched for a disease, the more disease cases that the province tended to report. However, the larger the number of per capita searches for a disease in a province, the fewer disease cases that the province tended to report. In addition, the search queries for the different categories tended to have different effects on the number of disease cases. For both the BSI and BSR, the category of treatment had a weaker influence than the other categories, while the category of prevention tended to have the greatest influence on the number of disease cases.

**Table 2:** Correlations between the Baidu search indexes (BSIs) and Baidu search rates (BSRs) of the 4 online health information-seeking behavior (OHISB) categories and the numbers of real-world cases of the 3 diseases.

Indicator, disease, and category			Model 1^a^		Model 2^b^		Model 3^c^		
			Coefficient, mean (SD)	*P* value	Coefficient, mean (SD)	*P* value	Coefficient, mean (SD)		*P* value
**BSI**									
	**Syphilis**								
		Concept	0.34 (0.03)	<.001	0.39 (0.03)	<.001	0.51 (0.04)		<.001
		Symptom	0.30 (0.03)	<.001	0.37 (0.04)	<.001	0.43 (0.04)		<.001
		Treatment	0.35 (0.03)	<.001	0.45 (0.04)	<.001	0.47 (0.05)		<.001
		Prevention	0.42 (0.04)	<.001	0.57 (0.05)	<.001	0.65 (0.06)		<.001
	**Gonorrhea**								
		Concept	0.40 (0.03)	<.001	0.46 (0.03)	<.001	0.45 (0.03)		<.001
		Symptom	0.48 (0.03)	<.001	0.50 (0.03	<.001	0.47 (0.03)		<.001
		Treatment	0.32 (0.03)	<.001	0.36 (0.03)	<.001	0.34 (0.03)		<.001
		Prevention	0.55 (0.05)	<.001	0.68 (0.06)	<.001	0.67 (0.06)		<.001
	**HIV/AIDS**								
		Concept	0.26 (0.04)	<.001	0.24 (0.05)	<.001	0.39 (0.05)		<.001
		Symptom	0.22 (0.04)	<.001	0.19 (0.04)	<.001	0.27 (0.04)		<.001
		Treatment	0.23 (0.03)	<.001	0.23 (0.04)	<.001	0.44 (0.04)		<.001
		Prevention	0.30 (0.04)	<.001	0.27 (0.05)	<.001	0.42 (0.06)		<.001
**BSR**									
	**Syphilis**								
		Concept	–0.07 (0.04)	.07	–0.08 (0.04)	.02	–0.33 (0.05)		<.001
		Symptom	–0.10 (0.03)	.002	–0.11 (0.03)	<.001	–0.25 (0.04)		<.001
		Treatment	–0.12 (0.04)	.001	–0.12 (0.04)	.002	–0.23 (0.04)		<.001
		Prevention	–0.17 (0.05)	.001	–0.17 (0.05)	<.001	–0.37 (0.06)		<.001
	**Gonorrhea**								
		Concept	0.05 (0.04)	.22	0.05 (0.04)	.17	–0.19 (0.05)		<.001
		Symptom	–0.01 (0.05)	.88	0.02 (0.04)	.65	–0.13 (0.05)		.01
		Treatment	–0.04 (0.03)	.28	–0.01 (0.03)	.70	–0.14 (0.04)		<.001
		Prevention	0.02 (0.06)	.73	0.02 (0.06)	.77	–0.28 (0.07)		<.001
	**HIV/AIDS**								
		Concept	–0.11 (0.04)	.004	–0.11 (0.04)	.003	–0.06 (0.05)		.21
		Symptom	–0.08 (0.04)	.04	–0.07 (0.04)	.045	–0.02 (0.04)		.69
		Treatment	–0.04 (0.04)	.32	–0.07 (0.04)	.06	0.03 (0.05)		.50
		Prevention	–0.16 (0.05)	.001	–0.14 (0.05)	.003	–0.09 (0.06)		.14

^a^Model 1 is the crude model without any confounders.

^b^Model 2 is the regression model adjusted for the male-female ratio and the proportion of the population over 65 years old.

^c^Model 3 is the regression model adjusted for the male-female ratio, the proportion of the population over 65 years old, the GRDP per capita, and the number of health institutions per capita.

### Time Lag Cross-Correlation Analyses

**Figure 2:**
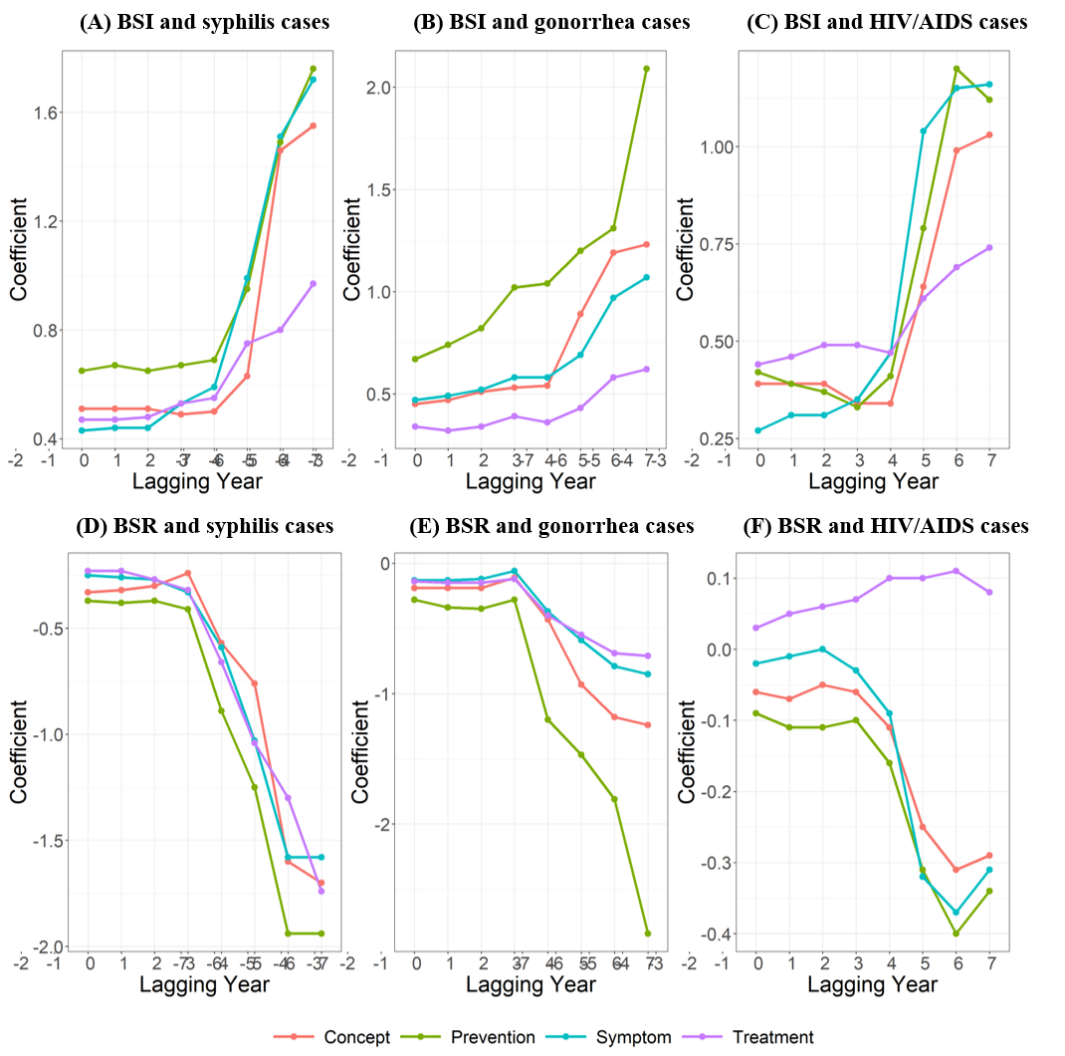
Time lag cross-correlations between BSIs and BSRs and numbers of real-world cases. (A) BSI and syphilis cases; (B) BSI and gonorrhea cases; (C) BSI and HIV/AIDS cases; (D) BSR and syphilis cases; (E) BSR and gonorrhea cases; (F) BSR and HIV/AIDS case. BSI: Baidu search index; BSR: Baidu search rate.

We also conducted time lag cross-correlation analyses using regression models. The coefficients of the model adjusted for GRDP per capita and the number of health institutions per capita is shown in Figure 2, and the specific regression coefficients with *P* values are presented in Table S3 in Multimedia Appendix 1. Except for the coefficient of BSR for the treatment category for HIV/AIDS, which did not achieve significance, all the coefficients were statistically significant (*P*<.05). As shown in Figure 2, the effect of the BSI and BSR on the number of disease cases increased with increasing lag years. Similar to the results above, prevention still had the highest impact, while treatment had the lowest impact over time. The coefficients of the other 2 models are shown in Figures S2 and S3 in Multimedia Appendix 1, and the specific regression coefficients with *P* values are shown in Tables S4 and S5 in Multimedia Appendix 1, with results similar to those in Figure 2. Besides, with the opposite direction of lagging years, we also ran it for sensitivity analysis, the result of which is shown in Figure S4 in Multimedia Appendix 1.

## Discussion

### Principal Results

Our study revealed geographical variability in both STD case numbers and OHISBs in China. We also found a complex association between OHISB and STD case numbers based on the Baidu index. Specifically, our results showed that for syphilis, gonorrhea, and HIV/AIDS, the BSIs were significantly positively correlated with case numbers, while the BSRs were significantly negatively correlated with case numbers. We also observed different levels of correlation among different types of OHISBs, including concept, prevention, symptom, and treatment search behaviors. Furthermore, we found that the impact of OHISB on the case number increased with the extension of the lag time.

Our study found notable geographical variability in the incidence of STDs in China. Consistent with previous studies, the incident case numbers of syphilis and gonorrhea were particularly high in the southeast region, especially in coastal provinces such as Guangdong and Zhejiang [25]. These areas experience high rates of migration, which increases the likelihood of sexually risky behaviors and facilitates the spread of STDs among prosperous regions [26]. On the other hand, HIV/AIDS was prevalent along the southeast border of China, where the high risk of drug trade and consumption leads to the easy transmission of the disease among residents in Yunnan, Sichuan, and Guangxi [27]. In addition, due to different cultural characteristics and customs, inadequate sexual and reproductive knowledge, and scarce medical resources, provinces in the northwest region like Xinjiang also had a relatively higher incidence of these STDs [28]. We also found that health information-seeking behavior in China displayed a pro-rich pattern, which could contribute to health literacy inequality [29]. Our study highlights the need for reducing inequities in the availability of health information to prevent unequal burdens of diseases [30]. However, efforts in this area in China are still in the early stages.

Previous studies have established a positive relationship between the BSI and STD case numbers [31]. With the increasing accessibility and real-time availability of internet-based health information, individuals at risk of STDs tend to seek high-quality health information on the internet in the early stages of the disease due to social discrimination and disease-related stigma [32,33]. This may explain why the number of newly diagnosed cases exhibited a strong positive correlation with the relative BSI.

On the contrary, this study is the first to explore the association between BSR and the number of disease cases. While the BSI can directly reflect the incident case number since it is calculated based on personal search behavior, a higher BSR indicates greater exposure to health information from a public perspective. According to the information-motivation-behavior skills model, health information is a prerequisite for behavioral change [34]. Therefore, as the BSR increases in a specific region, more preventive behaviors are likely to be adopted by residents [35]. Specifically, for syphilis, gonorrhea, and HIV/AIDS, internet users have access to abundant health information about transmission and prevention. An increased BSR indicates deeper and wider access to disease knowledge among the public, which can increase motivation to engage in STD risk reduction activities [36].

Patients and healthy people associated with patients were the 2 primary sources of web-based disease search queries [37]. Patients were also the major source of STD transmission to others, and from a public perspective, they had a relatively stable possibility of infecting others every year, as reflected by the increasingly positive association between case number and the BSI. Exposure to credible health information on the internet was associated with a higher level of health literacy [38], which could enhance the coping ability and sense of self-efficacy and facilitate communication of health behaviors to others, including improving the health behaviors of users and their friends and family and prompting discussions with health care providers [39,40]. Healthy people living with patients with STDs, as a high-risk group for STDs, are more likely to be directly impacted by the above effects of OHISB over time. As the BSR increases, residents in a region would have a deeper or wider understanding of STDs, which could improve their health literacy and lead to more long-term health behaviors. The effect of OHISB is reflected in the increasingly negative association between case number and BSR.

This study is novel in that it examines and compares the impact of different types of OHISBs on STD case numbers. According to the situational theory of problem-solving, an individual’s information-driven actions are affected by situational motivation in problem-solving [41]. Those who were newly diagnosed tended to conduct intensive searches for treatments and symptoms to better understand their condition, and those who were chronically ill generally searched for treatments to be discussed with their doctors. However, healthy people, especially those associated with patients with STD, who could be regarded as a high-risk group, tended to perform episodic searches about prevention. Since treatment-related searches were mostly performed by patients who had already been diagnosed, this behavior would result in a smaller impact on newly diagnosed cases. Prevention-related searches that focused on future diseases in healthy people who were at risk would result in a larger impact than the other types of searches.

### Limitations

There are some limitations to this study. First, the internet search query data we obtained from the Baidu index may be biased. Although Baidu is widely popular among Chinese people, the trend of users turning to social media for information seeking is increasing. Due to the lack of a uniform web-based recording system in China, it was difficult to obtain a series of representative internet search queries. However, it has been found that the Baidu index has better reliability for common diseases with minor coverage or rare diseases and conditions and a larger audience [42]. Second, noisy data existed in the Baidu index query. During some social informational campaigns or special festivals, such as World AIDS Day, STD search queries increase notably. Although we performed principal component analysis to account for multiple search words to minimize the impact of noise, it was difficult to eliminate. In addition, we used a year as a unit of time, and our time span was 8 years, from 2011 to 2018. Third, our search terms were not obtained from a systematic search strategy. They were chosen out of convenience and by subjective judgment. Thus, in the future, related studies should refine the unit of time to investigate the seasonal, monthly, or even daily effects of OHISB, lengthen the time span according to data availability, and develop a comprehensive and systematic search strategy. Fourth, though BSR indicated a deeper or wider exposure to information by residents, it is hard to distinguish these 2 mechanisms. Though we have controlled demographical structure and economic development as confounders, previous evidence showed that different regions tended to have similar internet search behaviors with a similar demographical structure, economic level, and adjacent regions [43,44]. It is better to be identified so that more targeted policies could be made. Fifth, disease outcomes and OHISB affected each other mutually. In this study, we only focused on the impact of OHISB on STDs. However, the opposite direction is also highly valued, especially the impact of infections on different types of OHISBs.

### Comparison With Prior Work

There are considerable mathematical, theoretical, and practical implications for this study. From a mathematical perspective, we used multisource big data, including real-world disease data, internet search query data, and socioeconomic and medical data, to investigate statistical associations. It is widely known that geographic internet use is greatly affected by economic conditions, just as health status is largely determined by local medical conditions. The combination of different types of data guaranteed the sensitivity of the study and produced more reliable results. From a theoretical perspective, by categorizing OHISBs into 4 types based on previous studies, we not only compared their impacts on disease outcomes but also investigated their long-term effects, both of which were analyzed for the first time to our knowledge. These results have great reference value regarding internet health information usage, health-seeking behavior, and disease transmission over time. From a practical perspective, this study revealed surveillance value, as demonstrated by the positive correlations between the case numbers of STDs and BSIs, as well as disease prevention value, as demonstrated by the negative correlation between the case numbers of STDs and BSRs. Accordingly, there is substantial potential for internet search query data to be extracted and applied to the public health field.

### Conclusions

OHISB had a significant association with the number of STD cases. Based on data from the Baidu search engine in China, different types of OHISBs had different impacts on diseases, and their impacts increased over time. These results provide a brand-new perspective for the potential applications of internet big data, including geographic surveillance as well as STD prevention.

## Appendix

Multimedia Appendix 1 Supplements.

## Abbreviations

BSI: Baidu search index
BSR: Baidu search rate
GRDP: gross regional domestic product
OHISB: online health information–seeking behavior
STD: sexually transmitted disease

## Data Availability

This study used publicly available repositories for analysis. The prevalence of 3 types of STDs can be obtained from the Chinese Center for Disease Control and Prevention [45] with a reasonable request. The economic index and health-related data can be obtained from the National Bureau of Statistics of China [46] through its statistical yearbook.
