# Supplementary material for: The Effects of Online Health Information–Seeking Behavior on Sexually Transmitted Disease in China: Infodemiology Study of the Internet Search Queries
Source: J Med Internet Res. 2023 May 12;25:e43046. doi: 10.2196/43046 (PMC10221515; doi:10.2196/43046)
Supplement: Multimedia Appendix 1 [file jmir_v25i1e43046_app1.pdf]

Multimedia Appendix 1

Table S1 The list of four economic regions and its provinces/municipalities in mainland China. ...2

Table S2 Search strategy of syphilis, gonorrhea, and HIV/AIDS in Baidu 3

Figure S1 the national trends of yearly numbers of confirmed cases of syphilis, gonorrhea, and HIV/AIDS in mainland China...5

Figure S2 Time-lag cross-correlations between BSIs and BSRs and numbers of crude model........6

Figure S3 Time-lag cross-correlations between BSIs and BSRs and numbers of the model adjusted for male-female ratio and proportion of population over 65 years old 7

Figure S4 Two-way time-lag cross-correlations between BSIs and BSRs and numbers of real-world cases 8

Table S3 the regression results of the model adjusted for male-female ratio, proportion of population over 65 years old, GRDP per capita and number of health institutions per capita...........................9

Table S4 the regression results of the crude model 14

Table S5 the regression results of the model adjusted for male-female ratio and proportion of population over 65 years old 19

Table S1 The list of four economic regions and its provinces/municipalities in mainland China.

**Economic region**	**Province/Municipality**
Eastern region	Beijing, Tianjin, Hebei, Shanghai, Jiangsu, Zhejiang, Fujian, Shandong, Guangdong, Hainan
Central region	Shanxi, Anhui, Jiangxi, Henan, Hubei, Hunan
Western region	Inner Mongolia, Guangxi, Chongqing, Sichuan, Guizhou, Yunnan, Tibet, Shaanxi, Gansu, Qinghai, Ningxia, Xinjiang
Northeastern region	Liaoning, Jilin, Heilongjiang

Table S2 Search strategy of syphilis, gonorrhea, and HIV/AIDS in Baidu

Disease	Category	Search term (Translated version)	Search term (Chinese)
Syphilis	Concept	syphilis (Chinese name)	梅毒
		syphilis (English name)	SYPHILIS
		primary syphilis	一期梅毒
		what is syphilis	梅毒是什么
	Symptom	symptoms and pictures of syphilis	梅毒的症状和图片
		symptoms of syphilis	梅毒症状
		pictures of syphilis	梅毒图片
		hard chancre	硬下疳
	Treatment	how to treat syphilis	梅毒怎么治疗
		syphilis treatment	梅毒治疗
		can syphilis be cured completely	梅毒可以彻底治愈吗
		can syphilis be cured	梅毒能治好吗
	Prevention	how is syphilis infected	梅毒怎么传染的
		incubation period of syphilis	梅毒潜伏期
		route of transmission of syphilis	梅毒传播途径
Gonorrhea	Concept	gonorrhea	淋病
		chronic gonorrhea	慢性淋病
		female gonorrhea	女性淋病
	Symptom	symptoms of gonorrhea	淋病症状
		pictures of gonorrhea	淋病图片
		what symptom does gonorrhea have	淋病有什么症状
		characteristics of gonorrhea	淋病的特征
	Treatment	what drug does gonorrhea take	淋病吃什么药
		the treatment of gonorrhea	淋病的治疗
		gonorrhea treatment	淋病治疗
		how is gonorrhea treated	淋病怎么治疗
		can gonorrhea be cured	淋病能治好吗
	Prevention	incubation period of gonorrhea	淋病潜伏期
HIV/AIDS	Concept	the disease of AIDS	艾滋病
		AIDS (Chinese name)	艾滋
		AIDS (English name)	AIDS
	Symptom	how long do you live with AIDS	艾滋病能活多久
		initial symptoms of AIDS	艾滋病初期症状
		symptoms of AIDS	艾滋症状
	Treatment	AIDS treatment	艾滋病治疗
		AIDS blocking drug	艾滋病阻断药
		AIDS cure	艾滋病治愈

	cocktail therapy	鸡尾酒疗法
Prevention	transmission of AIDS	艾滋病传播
	incubation period of AIDS	艾滋潜伏期
	route of transmission of AIDS	艾滋病传播途径
	prevention of AIDS	预防艾滋病
	AIDS prevention	艾滋病预防
	how to prevent AIDS	如何预防艾滋病

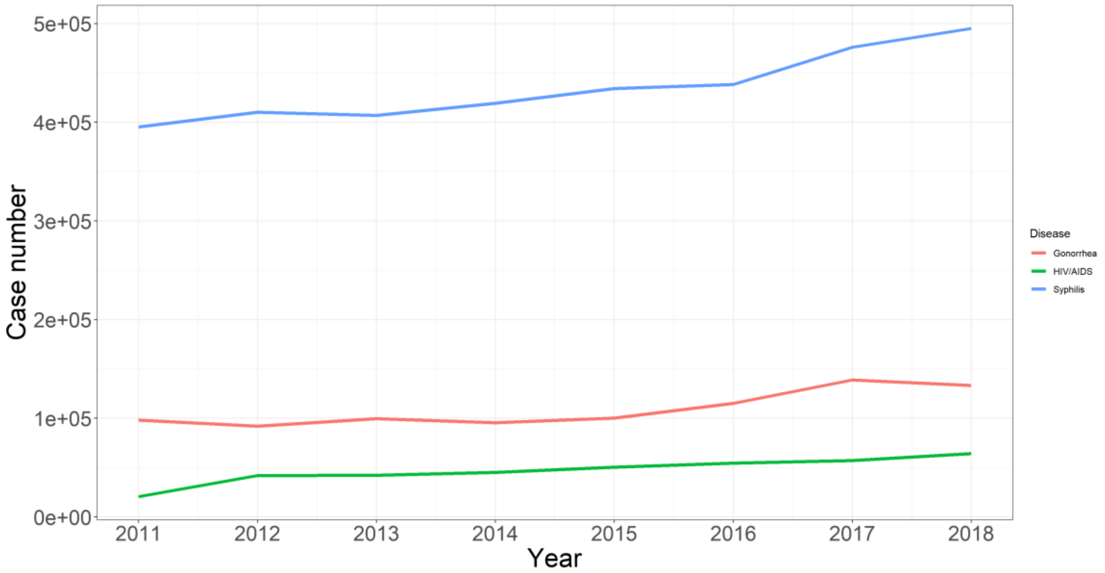

Figure S1 the national trends of yearly numbers of confirmed cases of syphilis, gonorrhea, and HIV/AIDS in mainland China

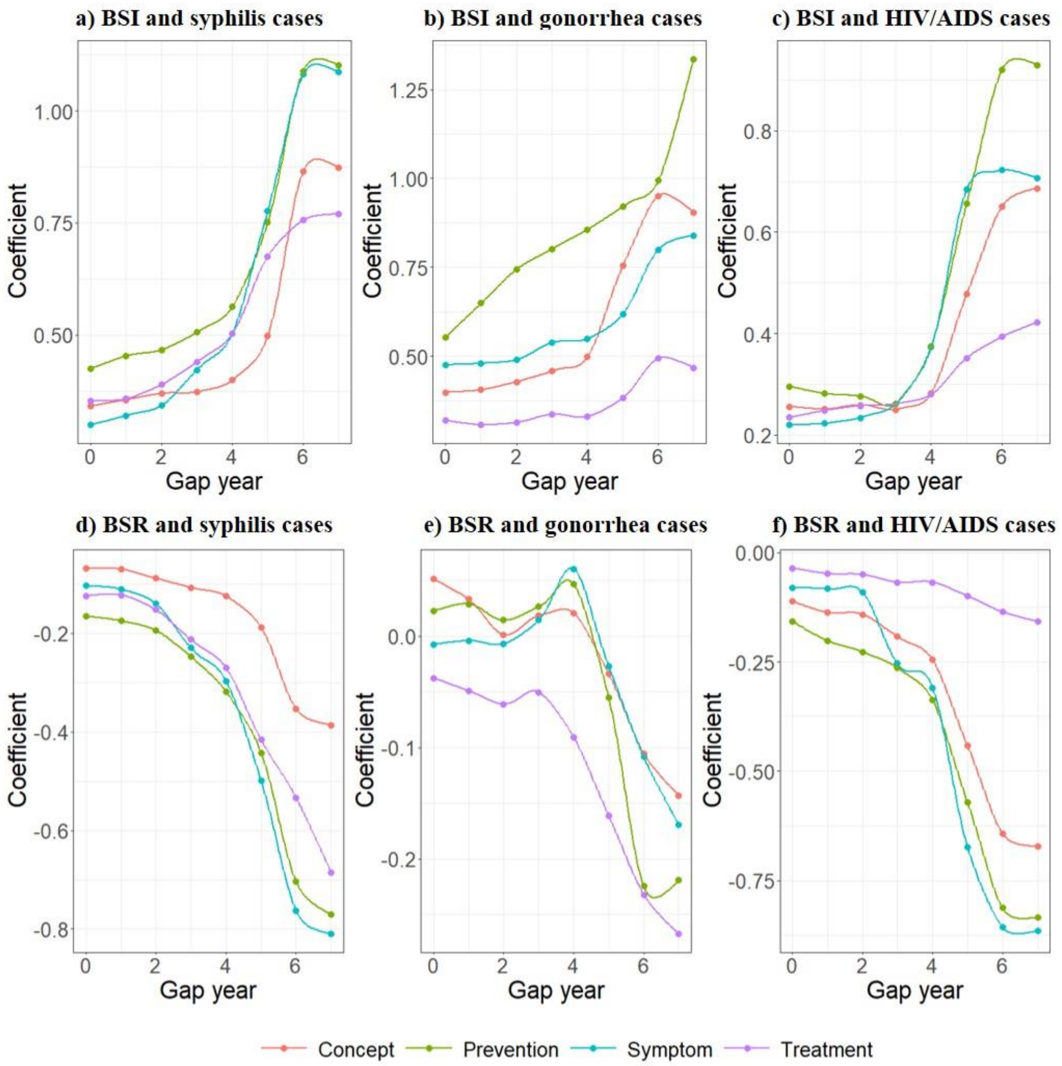

Figure S2 Time-lag cross-correlations between BSIs and BSRs and numbers of crude model

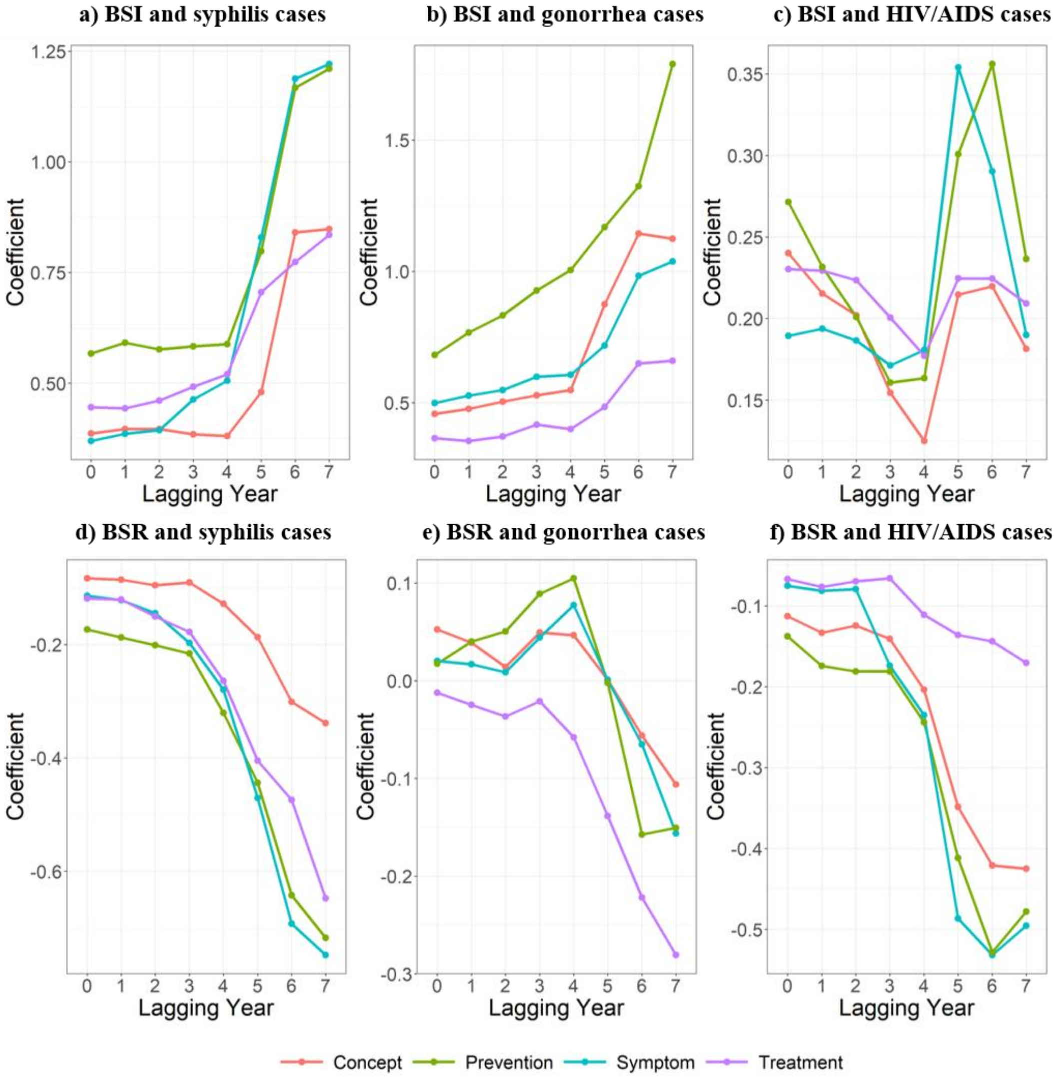

Figure S3 Time-lag cross-correlations between BSIs and BSRs and numbers of the model adjusted for male-female ratio and proportion of population over 65 years old

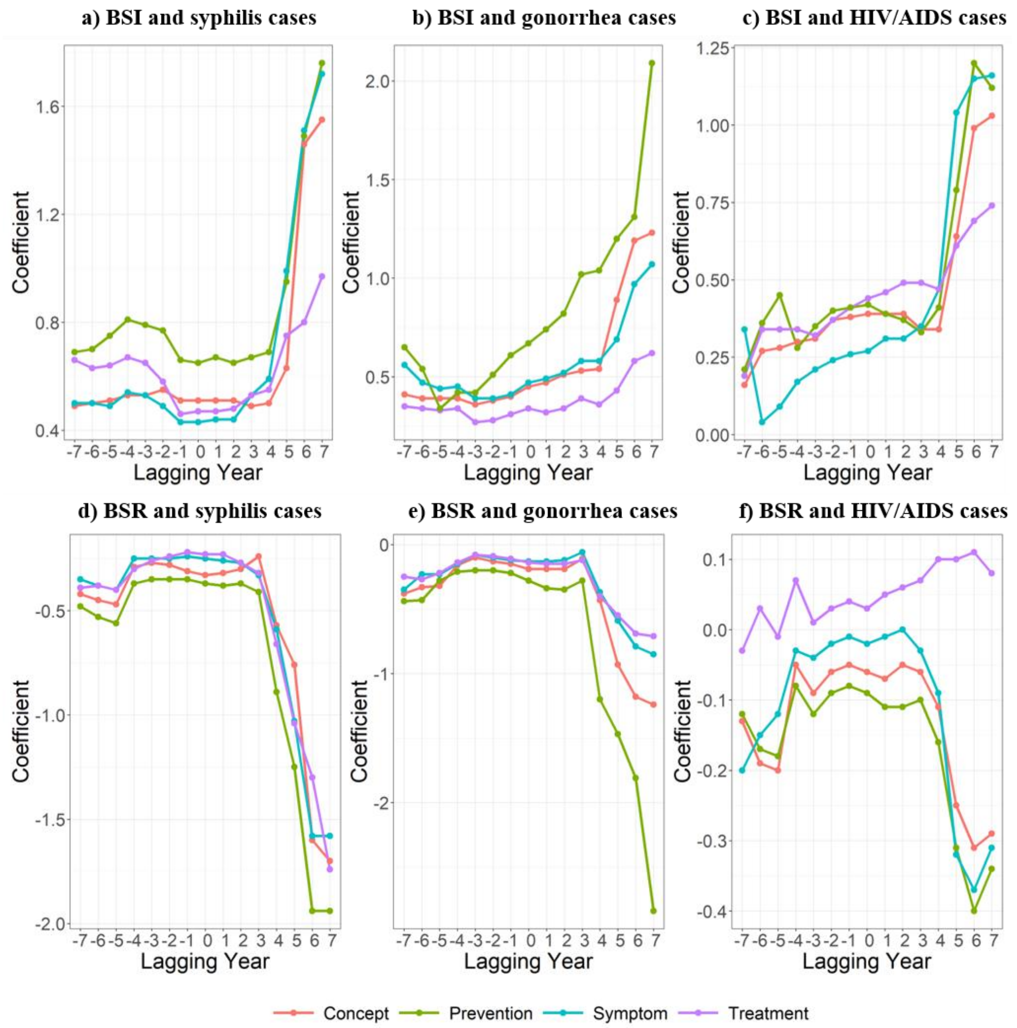

Figure S4 Two-way time-lag cross-correlations between BSIs and BSRs and numbers of real-world cases

Table S3 the regression results of the model adjusted for male-female ratio, proportion of population over 65 years old, GRDP per capita and number of health institutions per capita

Disease	Category	Index	Lagging year	Coefficient	Standard error	t_value	p_value
Syphilis	Concept	BSI	0	0.51	0.04	12.70	<.001
Syphilis	Concept	BSI	1	0.51	0.04	11.63	<.001
Syphilis	Concept	BSI	2	0.51	0.05	10.05	<.001
Syphilis	Concept	BSI	3	0.49	0.06	8.33	<.001
Syphilis	Concept	BSI	4	0.50	0.07	6.99	<.001
Syphilis	Concept	BSI	5	0.63	0.11	5.84	<.001
Syphilis	Concept	BSI	6	1.46	0.23	6.44	<.001
Syphilis	Concept	BSI	7	1.55	0.33	4.70	<.001
Syphilis	Concept	BSR	0	-0.33	0.05	-6.37	<.001
Syphilis	Concept	BSR	1	-0.32	0.06	-5.53	<.001
Syphilis	Concept	BSR	2	-0.30	0.06	-4.67	<.001
Syphilis	Concept	BSR	3	-0.24	0.07	-3.36	.001
Syphilis	Concept	BSR	4	-0.57	0.09	-6.48	<.001
Syphilis	Concept	BSR	5	-0.76	0.12	-6.12	<.001
Syphilis	Concept	BSR	6	-1.60	0.22	-7.19	<.001
Syphilis	Concept	BSR	7	-1.70	0.33	-5.07	<.001
Syphilis	Prevention	BSI	0	0.65	0.06	11.37	<.001
Syphilis	Prevention	BSI	1	0.67	0.06	10.35	<.001
Syphilis	Prevention	BSI	2	0.65	0.07	8.78	<.001
Syphilis	Prevention	BSI	3	0.67	0.09	7.37	<.001
Syphilis	Prevention	BSI	4	0.69	0.11	6.35	<.001
Syphilis	Prevention	BSI	5	0.95	0.17	5.75	<.001
Syphilis	Prevention	BSI	6	1.49	0.28	5.27	<.001
Syphilis	Prevention	BSI	7	1.76	0.45	3.87	.001
Syphilis	Prevention	BSR	0	-0.37	0.06	-6.55	<.001
Syphilis	Prevention	BSR	1	-0.38	0.06	-5.91	<.001
Syphilis	Prevention	BSR	2	-0.37	0.07	-5.06	<.001
Syphilis	Prevention	BSR	3	-0.41	0.10	-3.91	<.001
Syphilis	Prevention	BSR	4	-0.89	0.12	-7.42	<.001
Syphilis	Prevention	BSR	5	-1.25	0.17	-7.50	<.001
Syphilis	Prevention	BSR	6	-1.94	0.27	-7.29	<.001
Syphilis	Prevention	BSR	7	-1.94	0.37	-5.27	<.001
Syphilis	Symptom	BSI	0	0.43	0.04	10.43	<.001
Syphilis	Symptom	BSI	1	0.44	0.05	9.63	<.001
Syphilis	Symptom	BSI	2	0.44	0.05	8.13	<.001
Syphilis	Symptom	BSI	3	0.53	0.08	6.99	<.001
Syphilis	Symptom	BSI	4	0.59	0.10	6.00	<.001
Syphilis	Symptom	BSI	5	0.99	0.16	6.05	<.001
Syphilis	Symptom	BSI	6	1.51	0.30	5.02	<.001

Syphilis	Symptom	BSI	7	1.72	0.47	3.65	.001
Syphilis	Symptom	BSR	0	-0.25	0.04	-6.39	<.001
Syphilis	Symptom	BSR	1	-0.26	0.05	-5.72	<.001
Syphilis	Symptom	BSR	2	-0.27	0.05	-5.07	<.001
Syphilis	Symptom	BSR	3	-0.33	0.08	-4.26	<.001
Syphilis	Symptom	BSR	4	-0.59	0.08	-6.91	<.001
Syphilis	Symptom	BSR	5	-1.03	0.13	-7.79	<.001
Syphilis	Symptom	BSR	6	-1.58	0.20	-7.96	<.001
Syphilis	Symptom	BSR	7	-1.58	0.28	-5.55	<.001
Syphilis	Treatment	BSI	0	0.47	0.05	10.50	<.001
Syphilis	Treatment	BSI	1	0.47	0.05	9.61	<.001
Syphilis	Treatment	BSI	2	0.48	0.06	8.37	<.001
Syphilis	Treatment	BSI	3	0.53	0.07	7.08	<.001
Syphilis	Treatment	BSI	4	0.55	0.09	6.20	<.001
Syphilis	Treatment	BSI	5	0.75	0.14	5.47	<.001
Syphilis	Treatment	BSI	6	0.80	0.20	3.95	<.001
Syphilis	Treatment	BSI	7	0.97	0.30	3.22	.004
Syphilis	Treatment	BSR	0	-0.23	0.04	-5.18	<.001
Syphilis	Treatment	BSR	1	-0.23	0.05	-4.59	<.001
Syphilis	Treatment	BSR	2	-0.27	0.06	-4.12	<.001
Syphilis	Treatment	BSR	3	-0.32	0.10	-3.25	.001
Syphilis	Treatment	BSR	4	-0.66	0.12	-5.72	<.001
Syphilis	Treatment	BSR	5	-1.04	0.17	-6.02	<.001
Syphilis	Treatment	BSR	6	-1.30	0.27	-4.84	<.001
Syphilis	Treatment	BSR	7	-1.74	0.33	-5.29	<.001
Gonorrhoea	Concept	BSI	0	0.45	0.03	13.84	<.001
Gonorrhoea	Concept	BSI	1	0.47	0.04	12.91	<.001
Gonorrhoea	Concept	BSI	2	0.51	0.04	12.33	<.001
Gonorrhoea	Concept	BSI	3	0.53	0.05	11.21	<.001
Gonorrhoea	Concept	BSI	4	0.54	0.06	8.64	<.001
Gonorrhoea	Concept	BSI	5	0.89	0.11	8.19	<.001
Gonorrhoea	Concept	BSI	6	1.19	0.19	6.36	<.001
Gonorrhoea	Concept	BSI	7	1.23	0.27	4.61	<.001
Gonorrhoea	Concept	BSR	0	-0.19	0.05	-3.54	<.001
Gonorrhoea	Concept	BSR	1	-0.19	0.06	-3.44	.001
Gonorrhoea	Concept	BSR	2	-0.19	0.06	-3.10	.002
Gonorrhoea	Concept	BSR	3	-0.11	0.07	-1.52	.13
Gonorrhoea	Concept	BSR	4	-0.43	0.10	-4.42	<.001
Gonorrhoea	Concept	BSR	5	-0.93	0.17	-5.52	<.001
Gonorrhoea	Concept	BSR	6	-1.18	0.25	-4.66	<.001
Gonorrhoea	Concept	BSR	7	-1.24	0.37	-3.36	.002
Gonorrhoea	Prevention	BSI	0	0.67	0.06	10.37	<.001
Gonorrhoea	Prevention	BSI	1	0.74	0.08	9.46	<.001
Gonorrhoea	Prevention	BSI	2	0.82	0.09	8.83	<.001

Gonorrhoea	Prevention	BSI	3	1.02	0.11	9.58	<.001
Gonorrhoea	Prevention	BSI	4	1.04	0.13	8.07	<.001
Gonorrhoea	Prevention	BSI	5	1.20	0.18	6.52	<.001
Gonorrhoea	Prevention	BSI	6	1.31	0.31	4.24	<.001
Gonorrhoea	Prevention	BSI	7	2.09	0.57	3.66	.001
Gonorrhoea	Prevention	BSR	0	-0.28	0.07	-4.05	<.001
Gonorrhoea	Prevention	BSR	1	-0.34	0.10	-3.61	<.001
Gonorrhoea	Prevention	BSR	2	-0.35	0.12	-2.95	.004
Gonorrhoea	Prevention	BSR	3	-0.28	0.14	-2.03	.04
Gonorrhoea	Prevention	BSR	4	-1.20	0.20	-5.96	<.001
Gonorrhoea	Prevention	BSR	5	-1.47	0.25	-5.81	<.001
Gonorrhoea	Prevention	BSR	6	-1.81	0.35	-5.21	<.001
Gonorrhoea	Prevention	BSR	7	-2.84	0.68	-4.19	<.001
Gonorrhoea	Symptom	BSI	0	0.47	0.03	15.32	<.001
Gonorrhoea	Symptom	BSI	1	0.49	0.03	15.19	<.001
Gonorrhoea	Symptom	BSI	2	0.52	0.03	14.83	<.001
Gonorrhoea	Symptom	BSI	3	0.58	0.04	15.93	<.001
Gonorrhoea	Symptom	BSI	4	0.58	0.05	12.83	<.001
Gonorrhoea	Symptom	BSI	5	0.69	0.07	9.45	<.001
Gonorrhoea	Symptom	BSI	6	0.97	0.12	7.79	<.001
Gonorrhoea	Symptom	BSI	7	1.07	0.18	5.90	<.001
Gonorrhoea	Symptom	BSR	0	-0.13	0.05	-2.61	.01
Gonorrhoea	Symptom	BSR	1	-0.13	0.06	-2.37	.02
Gonorrhoea	Symptom	BSR	2	-0.12	0.07	-1.87	.06
Gonorrhoea	Symptom	BSR	3	-0.06	0.07	-0.82	.41
Gonorrhoea	Symptom	BSR	4	-0.37	0.11	-3.33	.001
Gonorrhoea	Symptom	BSR	5	-0.59	0.14	-4.05	<.001
Gonorrhoea	Symptom	BSR	6	-0.79	0.21	-3.72	<.001
Gonorrhoea	Symptom	BSR	7	-0.85	0.31	-2.74	.01
Gonorrhoea	Treatment	BSI	0	0.34	0.03	12.16	<.001
Gonorrhoea	Treatment	BSI	1	0.32	0.03	10.11	<.001
Gonorrhoea	Treatment	BSI	2	0.34	0.04	9.54	<.001
Gonorrhoea	Treatment	BSI	3	0.39	0.04	9.96	<.001
Gonorrhoea	Treatment	BSI	4	0.36	0.05	7.40	<.001
Gonorrhoea	Treatment	BSI	5	0.43	0.08	5.64	<.001
Gonorrhoea	Treatment	BSI	6	0.58	0.13	4.61	<.001
Gonorrhoea	Treatment	BSI	7	0.62	0.19	3.24	.003
Gonorrhoea	Treatment	BSR	0	-0.14	0.04	-3.89	<.001
Gonorrhoea	Treatment	BSR	1	-0.15	0.04	-3.82	<.001
Gonorrhoea	Treatment	BSR	2	-0.15	0.04	-3.37	.001
Gonorrhoea	Treatment	BSR	3	-0.12	0.05	-2.55	.01
Gonorrhoea	Treatment	BSR	4	-0.40	0.06	-6.31	<.001
Gonorrhoea	Treatment	BSR	5	-0.55	0.08	-6.55	<.001
Gonorrhoea	Treatment	BSR	6	-0.69	0.13	-5.45	<.001

Gonorrhoea	Treatment	BSR	7	-0.71	0.18	-3.95	.001
HIV/AIDS	Concept	BSI	0	0.39	0.05	7.84	<.001
HIV/AIDS	Concept	BSI	1	0.39	0.06	6.94	<.001
HIV/AIDS	Concept	BSI	2	0.39	0.06	6.50	<.001
HIV/AIDS	Concept	BSI	3	0.34	0.07	4.84	<.001
HIV/AIDS	Concept	BSI	4	0.34	0.09	3.67	<.001
HIV/AIDS	Concept	BSI	5	0.64	0.18	3.64	<.001
HIV/AIDS	Concept	BSI	6	0.99	0.36	2.76	.008
HIV/AIDS	Concept	BSI	7	1.03	0.60	1.71	.099
HIV/AIDS	Concept	BSR	0	-0.06	0.05	-1.26	.21
HIV/AIDS	Concept	BSR	1	-0.07	0.05	-1.22	.22
HIV/AIDS	Concept	BSR	2	-0.05	0.06	-0.80	.42
HIV/AIDS	Concept	BSR	3	-0.06	0.07	-0.78	.44
HIV/AIDS	Concept	BSR	4	-0.11	0.08	-1.34	.18
HIV/AIDS	Concept	BSR	5	-0.25	0.15	-1.70	.09
HIV/AIDS	Concept	BSR	6	-0.31	0.23	-1.33	.19
HIV/AIDS	Concept	BSR	7	-0.29	0.37	-0.78	.44
HIV/AIDS	Prevention	BSI	0	0.42	0.06	7.39	<.001
HIV/AIDS	Prevention	BSI	1	0.39	0.06	6.09	<.001
HIV/AIDS	Prevention	BSI	2	0.37	0.07	5.29	<.001
HIV/AIDS	Prevention	BSI	3	0.33	0.08	4.37	<.001
HIV/AIDS	Prevention	BSI	4	0.41	0.12	3.39	.001
HIV/AIDS	Prevention	BSI	5	0.79	0.23	3.35	.001
HIV/AIDS	Prevention	BSI	6	1.20	0.46	2.60	.01
HIV/AIDS	Prevention	BSI	7	1.12	0.78	1.43	.17
HIV/AIDS	Prevention	BSR	0	-0.09	0.06	-1.49	.14
HIV/AIDS	Prevention	BSR	1	-0.11	0.07	-1.66	.098
HIV/AIDS	Prevention	BSR	2	-0.11	0.08	-1.46	.15
HIV/AIDS	Prevention	BSR	3	-0.10	0.09	-1.22	.23
HIV/AIDS	Prevention	BSR	4	-0.16	0.09	-1.68	.095
HIV/AIDS	Prevention	BSR	5	-0.31	0.16	-1.97	.052
HIV/AIDS	Prevention	BSR	6	-0.40	0.26	-1.57	.12
HIV/AIDS	Prevention	BSR	7	-0.34	0.42	-0.82	.42
HIV/AIDS	Symptom	BSI	0	0.27	0.04	6.57	<.001
HIV/AIDS	Symptom	BSI	1	0.31	0.04	7.17	<.001
HIV/AIDS	Symptom	BSI	2	0.31	0.05	6.46	<.001
HIV/AIDS	Symptom	BSI	3	0.35	0.07	4.96	<.001
HIV/AIDS	Symptom	BSI	4	0.47	0.12	4.04	<.001
HIV/AIDS	Symptom	BSI	5	1.04	0.24	4.33	<.001
HIV/AIDS	Symptom	BSI	6	1.15	0.40	2.86	.006
HIV/AIDS	Symptom	BSI	7	1.16	0.68	1.70	.10
HIV/AIDS	Symptom	BSR	0	-0.02	0.04	-0.40	.69
HIV/AIDS	Symptom	BSR	1	-0.01	0.05	-0.11	.91
HIV/AIDS	Symptom	BSR	2	0.00	0.06	0.06	.95

HIV/AIDS	Symptom	BSR	3	-0.03	0.10	-0.34	.73
HIV/AIDS	Symptom	BSR	4	-0.09	0.12	-0.77	.44
HIV/AIDS	Symptom	BSR	5	-0.32	0.23	-1.36	.18
HIV/AIDS	Symptom	BSR	6	-0.37	0.33	-1.12	.27
HIV/AIDS	Symptom	BSR	7	-0.31	0.51	-0.61	.55
HIV/AIDS	Treatment	BSI	0	0.44	0.04	10.51	<.001
HIV/AIDS	Treatment	BSI	1	0.46	0.05	9.96	<.001
HIV/AIDS	Treatment	BSI	2	0.49	0.05	10.03	<.001
HIV/AIDS	Treatment	BSI	3	0.49	0.06	8.71	<.001
HIV/AIDS	Treatment	BSI	4	0.47	0.07	6.98	<.001
HIV/AIDS	Treatment	BSI	5	0.61	0.10	6.33	<.001
HIV/AIDS	Treatment	BSI	6	0.69	0.16	4.45	<.001
HIV/AIDS	Treatment	BSI	7	0.74	0.27	2.70	.01
HIV/AIDS	Treatment	BSR	0	0.03	0.05	0.68	.50
HIV/AIDS	Treatment	BSR	1	0.05	0.05	0.95	.35
HIV/AIDS	Treatment	BSR	2	0.06	0.06	1.14	.26
HIV/AIDS	Treatment	BSR	3	0.07	0.06	1.12	.26
HIV/AIDS	Treatment	BSR	4	0.10	0.10	1.07	.29
HIV/AIDS	Treatment	BSR	5	0.10	0.13	0.79	.43
HIV/AIDS	Treatment	BSR	6	0.11	0.19	0.56	.58
HIV/AIDS	Treatment	BSR	7	0.08	0.32	0.24	.81

Table S4 the regression results of the crude model

Disease	Category	Index	Lagging year	Coefficient	Standard error	t_value	p_value
Syphilis	Concept	BSI	0	0.34	0.03	12.23	<.001
Syphilis	Concept	BSI	1	0.36	0.03	11.42	<.001
Syphilis	Concept	BSI	2	0.37	0.04	10.27	<.001
Syphilis	Concept	BSI	3	0.37	0.04	8.87	<.001
Syphilis	Concept	BSI	4	0.40	0.05	7.53	<.001
Syphilis	Concept	BSI	5	0.50	0.08	6.18	<.001
Syphilis	Concept	BSI	6	0.86	0.16	5.50	<.001
Syphilis	Concept	BSI	7	0.87	0.23	3.88	.001
Syphilis	Concept	BSR	0	-0.07	0.04	-1.80	.07
Syphilis	Concept	BSR	1	-0.07	0.04	-1.64	.10
Syphilis	Concept	BSR	2	-0.09	0.05	-1.78	.07
Syphilis	Concept	BSR	3	-0.11	0.06	-1.83	.07
Syphilis	Concept	BSR	4	-0.12	0.07	-1.84	.07
Syphilis	Concept	BSR	5	-0.19	0.10	-1.96	.053
Syphilis	Concept	BSR	6	-0.35	0.17	-2.13	.04
Syphilis	Concept	BSR	7	-0.39	0.24	-1.62	.12
Syphilis	Prevention	BSI	0	0.42	0.04	10.91	<.001
Syphilis	Prevention	BSI	1	0.45	0.04	10.19	<.001
Syphilis	Prevention	BSI	2	0.47	0.05	9.12	<.001
Syphilis	Prevention	BSI	3	0.51	0.06	8.09	<.001
Syphilis	Prevention	BSI	4	0.56	0.08	7.05	<.001
Syphilis	Prevention	BSI	5	0.75	0.12	6.24	<.001
Syphilis	Prevention	BSI	6	1.09	0.20	5.48	<.001
Syphilis	Prevention	BSI	7	1.10	0.29	3.80	.001
Syphilis	Prevention	BSR	0	-0.17	0.05	-3.44	.001
Syphilis	Prevention	BSR	1	-0.17	0.06	-3.09	.002
Syphilis	Prevention	BSR	2	-0.19	0.07	-2.96	.003
Syphilis	Prevention	BSR	3	-0.25	0.09	-2.72	.007
Syphilis	Prevention	BSR	4	-0.32	0.11	-2.92	.004
Syphilis	Prevention	BSR	5	-0.44	0.15	-2.90	.005
Syphilis	Prevention	BSR	6	-0.70	0.24	-2.90	.005
Syphilis	Prevention	BSR	7	-0.77	0.33	-2.31	.03
Syphilis	Symptom	BSI	0	0.30	0.03	10.66	<.001
Syphilis	Symptom	BSI	1	0.32	0.03	10.04	<.001
Syphilis	Symptom	BSI	2	0.34	0.04	8.94	<.001
Syphilis	Symptom	BSI	3	0.42	0.05	8.05	<.001
Syphilis	Symptom	BSI	4	0.50	0.07	6.97	<.001
Syphilis	Symptom	BSI	5	0.78	0.12	6.53	<.001
Syphilis	Symptom	BSI	6	1.08	0.20	5.31	<.001
Syphilis	Symptom	BSI	7	1.09	0.29	3.70	.001
Syphilis	Symptom	BSR	0	-0.10	0.03	-3.13	.002

Syphilis	Symptom	BSR	1	-0.11	0.04	-2.85	.005
Syphilis	Symptom	BSR	2	-0.14	0.05	-2.96	.003
Syphilis	Symptom	BSR	3	-0.23	0.07	-3.34	.001
Syphilis	Symptom	BSR	4	-0.30	0.08	-3.65	<.001
Syphilis	Symptom	BSR	5	-0.50	0.13	-3.87	<.001
Syphilis	Symptom	BSR	6	-0.76	0.20	-3.82	<.001
Syphilis	Symptom	BSR	7	-0.81	0.28	-2.93	.007
Syphilis	Treatment	BSI	0	0.35	0.03	10.71	<.001
Syphilis	Treatment	BSI	1	0.36	0.04	9.95	<.001
Syphilis	Treatment	BSI	2	0.39	0.04	9.07	<.001
Syphilis	Treatment	BSI	3	0.44	0.05	8.02	<.001
Syphilis	Treatment	BSI	4	0.50	0.07	7.01	<.001
Syphilis	Treatment	BSI	5	0.68	0.11	6.16	<.001
Syphilis	Treatment	BSI	6	0.76	0.16	4.61	<.001
Syphilis	Treatment	BSI	7	0.77	0.22	3.58	.001
Syphilis	Treatment	BSR	0	-0.12	0.04	-3.24	.001
Syphilis	Treatment	BSR	1	-0.12	0.04	-2.79	.006
Syphilis	Treatment	BSR	2	-0.15	0.06	-2.66	.009
Syphilis	Treatment	BSR	3	-0.21	0.09	-2.45	.02
Syphilis	Treatment	BSR	4	-0.27	0.10	-2.59	.01
Syphilis	Treatment	BSR	5	-0.42	0.16	-2.67	.009
Syphilis	Treatment	BSR	6	-0.53	0.23	-2.32	.02
Syphilis	Treatment	BSR	7	-0.69	0.30	-2.30	.02
Gonorrhoea	Concept	BSI	0	0.40	0.03	14.10	<.001
Gonorrhoea	Concept	BSI	1	0.41	0.03	12.91	<.001
Gonorrhoea	Concept	BSI	2	0.43	0.04	11.99	<.001
Gonorrhoea	Concept	BSI	3	0.46	0.04	11.01	<.001
Gonorrhoea	Concept	BSI	4	0.50	0.06	8.81	<.001
Gonorrhoea	Concept	BSI	5	0.75	0.10	7.85	<.001
Gonorrhoea	Concept	BSI	6	0.95	0.16	5.94	<.001
Gonorrhoea	Concept	BSI	7	0.90	0.22	4.10	<.001
Gonorrhoea	Concept	BSR	0	0.05	0.04	1.24	.22
Gonorrhoea	Concept	BSR	1	0.03	0.05	0.73	.48
Gonorrhoea	Concept	BSR	2	0.00	0.05	0.02	.98
Gonorrhoea	Concept	BSR	3	0.02	0.06	0.30	.76
Gonorrhoea	Concept	BSR	4	0.02	0.08	0.27	.79
Gonorrhoea	Concept	BSR	5	-0.03	0.13	-0.27	.79
Gonorrhoea	Concept	BSR	6	-0.11	0.19	-0.57	.57
Gonorrhoea	Concept	BSR	7	-0.14	0.26	-0.55	.58
Gonorrhoea	Prevention	BSI	0	0.55	0.05	10.44	<.001
Gonorrhoea	Prevention	BSI	1	0.65	0.07	9.85	<.001
Gonorrhoea	Prevention	BSI	2	0.74	0.08	9.36	<.001
Gonorrhoea	Prevention	BSI	3	0.80	0.09	9.01	<.001
Gonorrhoea	Prevention	BSI	4	0.86	0.11	7.95	<.001

Gonorrhoea	Prevention	BSI	5	0.92	0.15	6.16	<.001
Gonorrhoea	Prevention	BSI	6	0.99	0.24	4.12	<.001
Gonorrhoea	Prevention	BSI	7	1.34	0.41	3.24	.003
Gonorrhoea	Prevention	BSR	0	0.02	0.06	0.35	.73
Gonorrhoea	Prevention	BSR	1	0.03	0.08	0.34	.73
Gonorrhoea	Prevention	BSR	2	0.01	0.11	0.13	.89
Gonorrhoea	Prevention	BSR	3	0.03	0.12	0.22	.82
Gonorrhoea	Prevention	BSR	4	0.05	0.15	0.32	.75
Gonorrhoea	Prevention	BSR	5	-0.06	0.19	-0.29	.77
Gonorrhoea	Prevention	BSR	6	-0.22	0.28	-0.80	.43
Gonorrhoea	Prevention	BSR	7	-0.22	0.49	-0.45	.66
Gonorrhoea	Symptom	BSI	0	0.48	0.03	15.67	<.001
Gonorrhoea	Symptom	BSI	1	0.48	0.03	14.99	<.001
Gonorrhoea	Symptom	BSI	2	0.49	0.03	14.05	<.001
Gonorrhoea	Symptom	BSI	3	0.54	0.04	14.47	<.001
Gonorrhoea	Symptom	BSI	4	0.55	0.05	12.12	<.001
Gonorrhoea	Symptom	BSI	5	0.62	0.07	8.88	<.001
Gonorrhoea	Symptom	BSI	6	0.80	0.12	6.87	<.001
Gonorrhoea	Symptom	BSI	7	0.84	0.17	5.08	<.001
Gonorrhoea	Symptom	BSR	0	-0.01	0.05	-0.16	.88
Gonorrhoea	Symptom	BSR	1	0.00	0.05	-0.08	.93
Gonorrhoea	Symptom	BSR	2	-0.01	0.06	-0.12	.90
Gonorrhoea	Symptom	BSR	3	0.01	0.06	0.23	.82
Gonorrhoea	Symptom	BSR	4	0.06	0.09	0.65	.52
Gonorrhoea	Symptom	BSR	5	-0.03	0.12	-0.23	.82
Gonorrhoea	Symptom	BSR	6	-0.11	0.17	-0.64	.53
Gonorrhoea	Symptom	BSR	7	-0.17	0.24	-0.71	.48
Gonorrhoea	Treatment	BSI	0	0.32	0.03	11.92	<.001
Gonorrhoea	Treatment	BSI	1	0.31	0.03	10.11	<.001
Gonorrhoea	Treatment	BSI	2	0.31	0.03	9.35	<.001
Gonorrhoea	Treatment	BSI	3	0.34	0.04	9.13	<.001
Gonorrhoea	Treatment	BSI	4	0.33	0.05	7.27	<.001
Gonorrhoea	Treatment	BSI	5	0.38	0.07	5.59	<.001
Gonorrhoea	Treatment	BSI	6	0.49	0.11	4.44	<.001
Gonorrhoea	Treatment	BSI	7	0.47	0.16	2.98	.006
Gonorrhoea	Treatment	BSR	0	-0.04	0.03	-1.08	.28
Gonorrhoea	Treatment	BSR	1	-0.05	0.04	-1.30	.20
Gonorrhoea	Treatment	BSR	2	-0.06	0.04	-1.49	.14
Gonorrhoea	Treatment	BSR	3	-0.05	0.04	-1.15	.25
Gonorrhoea	Treatment	BSR	4	-0.09	0.06	-1.47	.14
Gonorrhoea	Treatment	BSR	5	-0.16	0.08	-1.96	.054
Gonorrhoea	Treatment	BSR	6	-0.23	0.12	-1.94	.06
Gonorrhoea	Treatment	BSR	7	-0.27	0.16	-1.67	.11
HIV/AIDS	Concept	BSI	0	0.26	0.04	6.63	<.001

HIV/AIDS	Concept	BSI	1	0.25	0.04	5.64	<.001
HIV/AIDS	Concept	BSI	2	0.26	0.05	5.28	<.001
HIV/AIDS	Concept	BSI	3	0.25	0.06	4.28	<.001
HIV/AIDS	Concept	BSI	4	0.28	0.08	3.48	.001
HIV/AIDS	Concept	BSI	5	0.48	0.15	3.13	.002
HIV/AIDS	Concept	BSI	6	0.65	0.29	2.26	.03
HIV/AIDS	Concept	BSI	7	0.69	0.44	1.55	.13
HIV/AIDS	Concept	BSR	0	-0.11	0.04	-2.87	.004
HIV/AIDS	Concept	BSR	1	-0.14	0.04	-3.10	.002
HIV/AIDS	Concept	BSR	2	-0.14	0.05	-2.83	.005
HIV/AIDS	Concept	BSR	3	-0.19	0.06	-3.11	.002
HIV/AIDS	Concept	BSR	4	-0.25	0.08	-3.23	.002
HIV/AIDS	Concept	BSR	5	-0.44	0.12	-3.54	.001
HIV/AIDS	Concept	BSR	6	-0.64	0.19	-3.38	.001
HIV/AIDS	Concept	BSR	7	-0.67	0.29	-2.28	.030
HIV/AIDS	Prevention	BSI	0	0.30	0.04	6.61	<.001
HIV/AIDS	Prevention	BSI	1	0.28	0.05	5.39	<.001
HIV/AIDS	Prevention	BSI	2	0.28	0.06	4.79	<.001
HIV/AIDS	Prevention	BSI	3	0.26	0.06	4.17	<.001
HIV/AIDS	Prevention	BSI	4	0.38	0.11	3.46	.001
HIV/AIDS	Prevention	BSI	5	0.66	0.21	3.15	.002
HIV/AIDS	Prevention	BSI	6	0.92	0.39	2.36	.02
HIV/AIDS	Prevention	BSI	7	0.93	0.61	1.52	.14
HIV/AIDS	Prevention	BSR	0	-0.16	0.05	-3.37	.001
HIV/AIDS	Prevention	BSR	1	-0.20	0.05	-3.71	<.001
HIV/AIDS	Prevention	BSR	2	-0.23	0.06	-3.57	<.001
HIV/AIDS	Prevention	BSR	3	-0.26	0.07	-3.61	<.001
HIV/AIDS	Prevention	BSR	4	-0.34	0.09	-3.82	<.001
HIV/AIDS	Prevention	BSR	5	-0.57	0.14	-4.06	<.001
HIV/AIDS	Prevention	BSR	6	-0.81	0.22	-3.73	<.001
HIV/AIDS	Prevention	BSR	7	-0.83	0.33	-2.51	.018
HIV/AIDS	Symptom	BSI	0	0.22	0.04	6.25	<.001
HIV/AIDS	Symptom	BSI	1	0.22	0.04	5.97	<.001
HIV/AIDS	Symptom	BSI	2	0.23	0.04	5.47	<.001
HIV/AIDS	Symptom	BSI	3	0.26	0.06	4.34	<.001
HIV/AIDS	Symptom	BSI	4	0.37	0.10	3.58	<.001
HIV/AIDS	Symptom	BSI	5	0.68	0.21	3.25	.002
HIV/AIDS	Symptom	BSI	6	0.72	0.35	2.09	.04
HIV/AIDS	Symptom	BSI	7	0.71	0.52	1.35	.19
HIV/AIDS	Symptom	BSR	0	-0.08	0.04	-2.08	.04
HIV/AIDS	Symptom	BSR	1	-0.08	0.04	-2.05	.04
HIV/AIDS	Symptom	BSR	2	-0.09	0.05	-1.88	.06
HIV/AIDS	Symptom	BSR	3	-0.25	0.09	-2.98	.003
HIV/AIDS	Symptom	BSR	4	-0.31	0.10	-3.00	.003

HIV/AIDS	Symptom	BSR	5	-0.67	0.18	-3.68	<.001
HIV/AIDS	Symptom	BSR	6	-0.86	0.25	-3.39	.001
HIV/AIDS	Symptom	BSR	7	-0.87	0.37	-2.32	.03
HIV/AIDS	Treatment	BSI	0	0.23	0.03	7.29	<.001
HIV/AIDS	Treatment	BSI	1	0.25	0.04	6.54	<.001
HIV/AIDS	Treatment	BSI	2	0.26	0.04	6.25	<.001
HIV/AIDS	Treatment	BSI	3	0.26	0.05	5.53	<.001
HIV/AIDS	Treatment	BSI	4	0.28	0.06	4.79	<.001
HIV/AIDS	Treatment	BSI	5	0.35	0.09	4.07	<.001
HIV/AIDS	Treatment	BSI	6	0.39	0.13	2.93	.005
HIV/AIDS	Treatment	BSI	7	0.42	0.21	2.01	.054
HIV/AIDS	Treatment	BSR	0	-0.04	0.04	-0.99	.32
HIV/AIDS	Treatment	BSR	1	-0.05	0.04	-1.14	.26
HIV/AIDS	Treatment	BSR	2	-0.05	0.05	-1.10	.27
HIV/AIDS	Treatment	BSR	3	-0.07	0.05	-1.28	.20
HIV/AIDS	Treatment	BSR	4	-0.07	0.06	-1.05	.30
HIV/AIDS	Treatment	BSR	5	-0.10	0.09	-1.11	.27
HIV/AIDS	Treatment	BSR	6	-0.14	0.13	-1.06	.29
HIV/AIDS	Treatment	BSR	7	-0.16	0.20	-0.78	.44

Table S5 the regression results of the model adjusted for male-female ratio and proportion of population over 65 years old

Disease	Category	Index	Lagging year	Coefficient	Standard error	t_value	p_value
Syphilis	Concept	BSI	0	0.39	0.03	11.61	<.001
Syphilis	Concept	BSI	1	0.40	0.04	10.85	<.001
Syphilis	Concept	BSI	2	0.40	0.04	9.57	<.001
Syphilis	Concept	BSI	3	0.38	0.05	8.05	<.001
Syphilis	Concept	BSI	4	0.38	0.06	6.62	<.001
Syphilis	Concept	BSI	5	0.48	0.09	5.56	<.001
Syphilis	Concept	BSI	6	0.84	0.17	5.05	<.001
Syphilis	Concept	BSI	7	0.85	0.24	3.46	.002
Syphilis	Concept	BSR	0	-0.08	0.04	-2.30	.02
Syphilis	Concept	BSR	1	-0.09	0.04	-2.07	.04
Syphilis	Concept	BSR	2	-0.10	0.05	-1.99	.048
Syphilis	Concept	BSR	3	-0.09	0.06	-1.60	.11
Syphilis	Concept	BSR	4	-0.13	0.06	-1.99	.049
Syphilis	Concept	BSR	5	-0.19	0.09	-2.02	.047
Syphilis	Concept	BSR	6	-0.30	0.16	-1.83	.07
Syphilis	Concept	BSR	7	-0.34	0.24	-1.40	.17
Syphilis	Prevention	BSI	0	0.57	0.05	11.29	<.001
Syphilis	Prevention	BSI	1	0.59	0.06	10.52	<.001
Syphilis	Prevention	BSI	2	0.58	0.06	9.08	<.001
Syphilis	Prevention	BSI	3	0.58	0.08	7.72	<.001
Syphilis	Prevention	BSI	4	0.59	0.09	6.47	<.001
Syphilis	Prevention	BSI	5	0.80	0.14	5.82	<.001
Syphilis	Prevention	BSI	6	1.17	0.23	5.14	<.001
Syphilis	Prevention	BSI	7	1.21	0.35	3.47	.002
Syphilis	Prevention	BSR	0	-0.17	0.05	-3.75	<.001
Syphilis	Prevention	BSR	1	-0.19	0.05	-3.44	.001
Syphilis	Prevention	BSR	2	-0.20	0.06	-3.18	.002
Syphilis	Prevention	BSR	3	-0.22	0.09	-2.43	.02
Syphilis	Prevention	BSR	4	-0.32	0.10	-3.09	.003
Syphilis	Prevention	BSR	5	-0.44	0.15	-3.00	.003
Syphilis	Prevention	BSR	6	-0.64	0.24	-2.67	.01
Syphilis	Prevention	BSR	7	-0.72	0.34	-2.12	.04
Syphilis	Symptom	BSI	0	0.37	0.04	10.34	<.001
Syphilis	Symptom	BSI	1	0.39	0.04	9.75	<.001
Syphilis	Symptom	BSI	2	0.39	0.05	8.45	<.001
Syphilis	Symptom	BSI	3	0.46	0.06	7.38	<.001
Syphilis	Symptom	BSI	4	0.51	0.08	6.16	<.001
Syphilis	Symptom	BSI	5	0.83	0.14	6.09	<.001
Syphilis	Symptom	BSI	6	1.19	0.24	4.96	<.001
Syphilis	Symptom	BSI	7	1.22	0.36	3.38	.002

Syphilis	Symptom	BSR	0	-0.11	0.03	-3.58	<.001
Syphilis	Symptom	BSR	1	-0.12	0.04	-3.26	.001
Syphilis	Symptom	BSR	2	-0.14	0.05	-3.18	.002
Syphilis	Symptom	BSR	3	-0.20	0.07	-2.91	.004
Syphilis	Symptom	BSR	4	-0.28	0.08	-3.61	<.001
Syphilis	Symptom	BSR	5	-0.47	0.13	-3.74	<.001
Syphilis	Symptom	BSR	6	-0.69	0.20	-3.46	.001
Syphilis	Symptom	BSR	7	-0.75	0.29	-2.62	.01
Syphilis	Treatment	BSI	0	0.45	0.04	10.86	<.001
Syphilis	Treatment	BSI	1	0.44	0.04	10.06	<.001
Syphilis	Treatment	BSI	2	0.46	0.05	8.94	<.001
Syphilis	Treatment	BSI	3	0.49	0.06	7.64	<.001
Syphilis	Treatment	BSI	4	0.52	0.08	6.54	<.001
Syphilis	Treatment	BSI	5	0.71	0.12	5.85	<.001
Syphilis	Treatment	BSI	6	0.77	0.18	4.36	<.001
Syphilis	Treatment	BSI	7	0.83	0.25	3.31	.003
Syphilis	Treatment	BSR	0	-0.12	0.04	-3.21	.002
Syphilis	Treatment	BSR	1	-0.12	0.04	-2.86	.005
Syphilis	Treatment	BSR	2	-0.15	0.06	-2.72	.007
Syphilis	Treatment	BSR	3	-0.18	0.09	-2.09	.04
Syphilis	Treatment	BSR	4	-0.26	0.10	-2.64	.009
Syphilis	Treatment	BSR	5	-0.40	0.15	-2.67	.009
Syphilis	Treatment	BSR	6	-0.47	0.23	-2.06	.04
Syphilis	Treatment	BSR	7	-0.65	0.30	-2.13	.04
Gonorrhoea	Concept	BSI	0	0.46	0.03	16.07	<.001
Gonorrhoea	Concept	BSI	1	0.48	0.03	15.14	<.001
Gonorrhoea	Concept	BSI	2	0.50	0.04	14.30	<.001
Gonorrhoea	Concept	BSI	3	0.53	0.04	13.04	<.001
Gonorrhoea	Concept	BSI	4	0.55	0.05	10.11	<.001
Gonorrhoea	Concept	BSI	5	0.87	0.09	9.42	<.001
Gonorrhoea	Concept	BSI	6	1.14	0.16	7.36	<.001
Gonorrhoea	Concept	BSI	7	1.12	0.22	5.11	<.001
Gonorrhoea	Concept	BSR	0	0.05	0.04	1.37	.17
Gonorrhoea	Concept	BSR	1	0.04	0.04	0.92	.36
Gonorrhoea	Concept	BSR	2	0.01	0.05	0.29	.77
Gonorrhoea	Concept	BSR	3	0.05	0.06	0.86	.39
Gonorrhoea	Concept	BSR	4	0.05	0.07	0.64	.53
Gonorrhoea	Concept	BSR	5	0.00	0.13	0.01	.99
Gonorrhoea	Concept	BSR	6	-0.06	0.19	-0.29	.77
Gonorrhoea	Concept	BSR	7	-0.11	0.28	-0.39	.70
Gonorrhoea	Prevention	BSI	0	0.68	0.06	12.25	<.001
Gonorrhoea	Prevention	BSI	1	0.77	0.07	11.42	<.001
Gonorrhoea	Prevention	BSI	2	0.83	0.08	10.63	<.001
Gonorrhoea	Prevention	BSI	3	0.93	0.09	10.71	<.001

Gonorrhoea	Prevention	BSI	4	1.01	0.10	9.83	<.001
Gonorrhoea	Prevention	BSI	5	1.17	0.15	7.79	<.001
Gonorrhoea	Prevention	BSI	6	1.32	0.25	5.26	<.001
Gonorrhoea	Prevention	BSI	7	1.79	0.43	4.14	<.001
Gonorrhoea	Prevention	BSR	0	0.02	0.06	0.29	.77
Gonorrhoea	Prevention	BSR	1	0.04	0.08	0.50	.62
Gonorrhoea	Prevention	BSR	2	0.05	0.10	0.50	.61
Gonorrhoea	Prevention	BSR	3	0.09	0.11	0.79	.43
Gonorrhoea	Prevention	BSR	4	0.10	0.14	0.75	.46
Gonorrhoea	Prevention	BSR	5	0.00	0.19	-0.01	.99
Gonorrhoea	Prevention	BSR	6	-0.16	0.29	-0.55	.58
Gonorrhoea	Prevention	BSR	7	-0.15	0.51	-0.30	.77
Gonorrhoea	Symptom	BSI	0	0.50	0.03	16.58	<.001
Gonorrhoea	Symptom	BSI	1	0.53	0.03	16.77	<.001
Gonorrhoea	Symptom	BSI	2	0.55	0.03	16.33	<.001
Gonorrhoea	Symptom	BSI	3	0.60	0.03	17.50	<.001
Gonorrhoea	Symptom	BSI	4	0.61	0.04	14.49	<.001
Gonorrhoea	Symptom	BSI	5	0.72	0.07	10.79	<.001
Gonorrhoea	Symptom	BSI	6	0.98	0.11	8.95	<.001
Gonorrhoea	Symptom	BSI	7	1.04	0.16	6.60	<.001
Gonorrhoea	Symptom	BSR	0	0.02	0.04	0.46	.65
Gonorrhoea	Symptom	BSR	1	0.02	0.05	0.35	.73
Gonorrhoea	Symptom	BSR	2	0.01	0.06	0.15	.88
Gonorrhoea	Symptom	BSR	3	0.04	0.06	0.73	.46
Gonorrhoea	Symptom	BSR	4	0.08	0.09	0.89	.38
Gonorrhoea	Symptom	BSR	5	0.00	0.12	0.01	.99
Gonorrhoea	Symptom	BSR	6	-0.07	0.18	-0.37	.72
Gonorrhoea	Symptom	BSR	7	-0.16	0.26	-0.59	.56
Gonorrhoea	Treatment	BSI	0	0.36	0.03	13.60	<.001
Gonorrhoea	Treatment	BSI	1	0.35	0.03	11.61	<.001
Gonorrhoea	Treatment	BSI	2	0.37	0.03	11.13	<.001
Gonorrhoea	Treatment	BSI	3	0.42	0.04	11.48	<.001
Gonorrhoea	Treatment	BSI	4	0.40	0.04	8.95	<.001
Gonorrhoea	Treatment	BSI	5	0.48	0.07	6.89	<.001
Gonorrhoea	Treatment	BSI	6	0.65	0.11	5.68	<.001
Gonorrhoea	Treatment	BSI	7	0.66	0.17	3.92	.001
Gonorrhoea	Treatment	BSR	0	-0.01	0.03	-0.38	.70
Gonorrhoea	Treatment	BSR	1	-0.02	0.04	-0.69	.49
Gonorrhoea	Treatment	BSR	2	-0.04	0.04	-0.93	.35
Gonorrhoea	Treatment	BSR	3	-0.02	0.04	-0.50	.62
Gonorrhoea	Treatment	BSR	4	-0.06	0.06	-0.98	.33
Gonorrhoea	Treatment	BSR	5	-0.14	0.08	-1.67	.099
Gonorrhoea	Treatment	BSR	6	-0.22	0.13	-1.74	.09
Gonorrhoea	Treatment	BSR	7	-0.28	0.18	-1.59	.12

HIV/AIDS	Concept	BSI	0	0.24	0.05	4.93	<.001
HIV/AIDS	Concept	BSI	1	0.22	0.05	3.93	<.001
HIV/AIDS	Concept	BSI	2	0.20	0.06	3.44	.001
HIV/AIDS	Concept	BSI	3	0.15	0.07	2.29	.02
HIV/AIDS	Concept	BSI	4	0.12	0.09	1.44	.15
HIV/AIDS	Concept	BSI	5	0.21	0.16	1.33	.19
HIV/AIDS	Concept	BSI	6	0.22	0.30	0.73	.47
HIV/AIDS	Concept	BSI	7	0.18	0.47	0.38	.71
HIV/AIDS	Concept	BSR	0	-0.11	0.04	-3.00	.003
HIV/AIDS	Concept	BSR	1	-0.13	0.04	-3.12	.002
HIV/AIDS	Concept	BSR	2	-0.12	0.05	-2.58	.01
HIV/AIDS	Concept	BSR	3	-0.14	0.06	-2.33	.02
HIV/AIDS	Concept	BSR	4	-0.20	0.07	-2.90	.004
HIV/AIDS	Concept	BSR	5	-0.35	0.12	-3.01	.003
HIV/AIDS	Concept	BSR	6	-0.42	0.19	-2.26	.03
HIV/AIDS	Concept	BSR	7	-0.43	0.30	-1.44	.16
HIV/AIDS	Prevention	BSI	0	0.27	0.05	4.94	<.001
HIV/AIDS	Prevention	BSI	1	0.23	0.06	3.71	<.001
HIV/AIDS	Prevention	BSI	2	0.20	0.07	3.01	.003
HIV/AIDS	Prevention	BSI	3	0.16	0.07	2.26	.03
HIV/AIDS	Prevention	BSI	4	0.16	0.12	1.40	.16
HIV/AIDS	Prevention	BSI	5	0.30	0.22	1.36	.18
HIV/AIDS	Prevention	BSI	6	0.36	0.41	0.88	.39
HIV/AIDS	Prevention	BSI	7	0.24	0.65	0.36	.72
HIV/AIDS	Prevention	BSR	0	-0.14	0.05	-3.02	.003
HIV/AIDS	Prevention	BSR	1	-0.17	0.05	-3.25	.001
HIV/AIDS	Prevention	BSR	2	-0.18	0.06	-2.87	.005
HIV/AIDS	Prevention	BSR	3	-0.18	0.07	-2.45	.02
HIV/AIDS	Prevention	BSR	4	-0.24	0.08	-2.88	.005
HIV/AIDS	Prevention	BSR	5	-0.41	0.14	-2.96	.004
HIV/AIDS	Prevention	BSR	6	-0.53	0.23	-2.29	.03
HIV/AIDS	Prevention	BSR	7	-0.48	0.37	-1.29	.21
HIV/AIDS	Symptom	BSI	0	0.19	0.04	4.69	<.001
HIV/AIDS	Symptom	BSI	1	0.19	0.04	4.39	<.001
HIV/AIDS	Symptom	BSI	2	0.19	0.05	3.80	<.001
HIV/AIDS	Symptom	BSI	3	0.17	0.07	2.54	.01
HIV/AIDS	Symptom	BSI	4	0.18	0.11	1.64	.10
HIV/AIDS	Symptom	BSI	5	0.35	0.22	1.61	.11
HIV/AIDS	Symptom	BSI	6	0.29	0.34	0.85	.40
HIV/AIDS	Symptom	BSI	7	0.19	0.54	0.35	.73
HIV/AIDS	Symptom	BSR	0	-0.07	0.04	-2.01	.045
HIV/AIDS	Symptom	BSR	1	-0.08	0.04	-2.08	.04
HIV/AIDS	Symptom	BSR	2	-0.08	0.05	-1.71	.09
HIV/AIDS	Symptom	BSR	3	-0.17	0.08	-2.07	.04

HIV/AIDS	Symptom	BSR	4	-0.24	0.10	-2.44	.02
HIV/AIDS	Symptom	BSR	5	-0.49	0.18	-2.77	.007
HIV/AIDS	Symptom	BSR	6	-0.53	0.26	-2.06	.04
HIV/AIDS	Symptom	BSR	7	-0.50	0.40	-1.25	.22
HIV/AIDS	Treatment	BSI	0	0.23	0.04	5.70	<.001
HIV/AIDS	Treatment	BSI	1	0.23	0.05	4.97	<.001
HIV/AIDS	Treatment	BSI	2	0.22	0.05	4.51	<.001
HIV/AIDS	Treatment	BSI	3	0.20	0.06	3.62	<.001
HIV/AIDS	Treatment	BSI	4	0.18	0.06	2.74	.007
HIV/AIDS	Treatment	BSI	5	0.22	0.09	2.46	.02
HIV/AIDS	Treatment	BSI	6	0.22	0.14	1.63	.11
HIV/AIDS	Treatment	BSI	7	0.21	0.23	0.93	.36
HIV/AIDS	Treatment	BSR	0	-0.07	0.04	-1.91	.06
HIV/AIDS	Treatment	BSR	1	-0.08	0.04	-1.87	.06
HIV/AIDS	Treatment	BSR	2	-0.07	0.04	-1.59	.11
HIV/AIDS	Treatment	BSR	3	-0.07	0.05	-1.32	.19
HIV/AIDS	Treatment	BSR	4	-0.11	0.06	-1.89	.06
HIV/AIDS	Treatment	BSR	5	-0.14	0.08	-1.71	.09
HIV/AIDS	Treatment	BSR	6	-0.14	0.11	-1.28	.21
HIV/AIDS	Treatment	BSR	7	-0.17	0.18	-0.94	.36
